# Bradycardia, Hyperkalemia, Renal Dysfunction, and Hypoglycemia in Guideline-Directed Medical Therapy for Heart Failure: When to Tolerate and When to Worry

**DOI:** 10.31083/RCM45741

**Published:** 2026-02-25

**Authors:** Maria Giulia Bellicini

**Affiliations:** ^1^Institute of Cardiology, ASST Spedali Civili di Brescia, 25123 Brescia, Italy

**Keywords:** heart failure, guideline-directed medical therapy, bradycardia, acute renal insufficiency, hyperkalemia, hypotension, hypoglycemia

## Abstract

A paradox persists in contemporary heart failure (HF) care, whereby the therapies most clearly proven to save the most lives are also those most frequently interrupted, often for reasons that are more physiological than pathological. Indeed, during HF medical therapy bradycardia, modest increases in creatinine or potassium levels, mild reductions in blood pressure, and concern regarding hypoglycemia are frequently perceived as dangerous adverse effects of drugs therapy, leading to premature dose reductions or discontinuation. However, when interpreted within their pharmacological and physiological context, these findings more often reflect predictable, dose-related drug effects rather than true toxicity. In the absence of predisposing conditions, such changes are typically modest in magnitude and unlikely to progress to clinically relevant pathological alterations. Recognizing these signals as expected manifestations of effective therapy, rather than harmful events, allows clinicians to maintain evidence-based drugs at target or near-target doses and to fully realize the mortality reduction associated with comprehensive guideline-directed medical therapy (GDMT).

## 1. Introduction

Despite the well-established efficacy of the four foundational classes of guideline-directed heart failure (HF) medical therapy (GDMT)—β-blockers, angiotensin-converting enzyme inhibitors (ACEi), angiotensin receptor blockers (ARB), or angiotensin receptor–neprilysin inhibitor (ARNI), mineralocorticoid receptor antagonists (MRA), and sodium–glucose cotransporter-2 inhibitors (SGLT2i)—therapeutic inertia remains a major contributor to preventable mortality. When appropriately initiated, up-titrated, and combined, these therapies reduce all-cause mortality by approximately [1, 2]. Nevertheless, a substantial proportion of patients remain undertreated in routine clinical practice [3, 4]. Bradycardia, hyperkalaemia, increases in serum creatinine, or reductions in plasma glucose are frequently misinterpreted as drug-related toxicity rather than anticipated physiological responses. This commentary reframes these findings through a pathophysiological and clinical perspective, delineating when treatment should be continued, reassessed, or discontinued.

## 2. Beta-Blockers and Bradycardia: Syncope Matters More Than Heart Rate

Beta (β)-blockers slow sinus rhythm and atrioventricular conduction by dampening sympathetic tone [5, 6]. Notably, this deceleration is modest and predictable in patients with an intact conduction system. Meanwhile, when no history of syncope is present, significant conduction disease, which constitutes an absolute contraindication, can be reasonably excluded, and a low resting pulse should not be considered hazardous. Minor conduction delays, such as first-degree or Wenckebach patterns, are often physiological modulations rather than pathology [7]. Therefore, dose titration should follow clinical tolerance (the appearance of syncope) rather than a numerical heart rate threshold: a patient who remains alert and asymptomatic can safely undergo further uptitration, even if the heart rate is below 50 bpm. Only true syncope warrants dose adjustment or investigation for underlying conduction disease [1].

How Can This Principle Be Applied in Daily Clinical Practice? For example initiation may begin with Bisoprolol 2.5 mg twice daily, indicating to the patient that, if well tolerated, the dose can be advanced to 5 mg twice daily. Meanwhile, a single 2.5 mg daily dose may represent an appropriate starting point for frail or older individuals.

## 3. ACE Inhibitors, ARBs, and ARNIs and Creatinine Rise: Final eGFR Matters More Than the Increase 

A mild-to-moderate rise in serum creatinine after administering an ACE inhibitor or ARB represents an expected hemodynamic effect of efferent arteriolar dilation, which lowers intraglomerular pressure and mildly reduces filtration [8, 9]. Similar, usually modest, changes in renal function may also be observed after initiating ARNI, largely reflecting the angiotensin receptor-blocking component of the drug rather than neprilysin inhibition [10]. Two complementary aspects should guide interpretation:

- The magnitude of the increase. An elevation of up to 50% from baseline is generally acceptable and reflects a physiological adaptation rather than injury [11, 12]. However, when the rise exceeds 50%, clinicians should suspect bilateral renal artery stenosis or another structural limitation to renal perfusion. In such cases, the issue is anatomical rather than pharmacological and is always accompanied by anuria/oliguria.
- The absolute filtration value after adaptation. What ultimately matters is the new steady-state of the estimated glomerular filtration rate (eGFR). If, after the change, the eGFR remains ≥25–30 mL/min/1.73 m^2^ (below this range the rate represents a contraindication), the kidney still filters several tens of milliliters per minute, sufficient for effective excretion and metabolic homeostasis [13]. At this level of filtration, renal autoregulation is preserved, and a continued angiotensin-converting enzyme inhibitor (RAAS) blockade remains safe and protective in the long term. Symptomatic hypotension should guide patient tolerance.

How Can This Principle Be Applied in Daily Clinical Practice? When baseline renal function is preserved (eGFR ≥30 mL/min/1.73 m^2^) and potassium is <5.0 mmol/L, ACE inhibitor or ARNI therapy can be safely initiated. For example, the starting dose of Ramipril should reflect the baseline blood pressure of the patient: up to 10 mg daily may be used in hypertensive patients, whereas lower doses are reasonable in those with borderline systolic values, with subsequent uptitration to the maximum tolerated dose according to blood pressure and symptoms of hypotension.

Transition to an ARNI, given the stronger vasodilatory and hypotensive effect of these inhibitors, should be considered only when the patient maintains adequate blood pressure even at the maximal tolerated dose of an ACE inhibitor or ARB.

## 4. Hyperkalemia: Renal Clearance Matters More Than Potassium Level in RAAS Inhibition

A kidney filtering above 30 mL/min/1.73 m^2^ can readily excrete potassium even under combined RAAS blockade (ACEi/ARBs and MRAs); homeostasis may remain preserved even below this threshold, and values below this level may still represent a contraindication [14, 15]. In such patients, reported high potassium levels are often due to pseudohyperkalemia, caused by sample hemolysis or delayed processing [16]. However, potassium levels up to 5.5 mmol/L are generally well-tolerated. In contrast, clinically relevant arrhythmic risk typically arises only when values exceed 6.0 mmol/L, which represents an absolute contraindication to potassium-raising drugs, a level that occurs normally only in the presence of acidosis or advanced renal dysfunction [17]. When persistent or recurrent hyperkalemia limits the continuation of MRA (or in general RAAS inhibitors), new-generation potassium binders, such as patiromer or sodium zirconium cyclosilicate, can be used to maintain therapy safely [18].

Landmark trials (RALES, EPHESUS, EMPHASIS-HF) showed that severe hyperkalemia was uncommon when baseline renal function was adequate [19, 20, 21]; mild-to-moderate potassium elevations (≤5.5 mmol/L) were common but rarely led to treatment discontinuation or adverse outcomes, confirming that such increases are generally benign when renal function is preserved. More recent studies with finerenone for diabetic kidney disease (FIDELIO-DKD, FIGARO-DKD) confirmed that even high-risk patients could be managed safely with appropriate monitoring [22, 23].

How Can This Principle Be Applied in Daily Clinical Practice? MRAs can usually be started directly at the target dose for patients with an eGFR ≥30 mL/min/1.73 m^2^ and serum potassium <5.0 mmol/L.

## 5. Hypoglycemia, Hypotension, Creatinine Increase, and SGLT2is: The Safest Pillar of Guideline-Directed Therapy

SGLT2i lowers plasma glucose by enhancing urinary glucose excretion, but only when blood glucose levels exceed the renal threshold for glucose excretion, which is shifted into the normoglycemic range (≈70–90 mg/dL) [24].

If glucose levels are normal, the filtered load of glucose is small, and these agents simply do not act; there is no further glycosuria and thus no risk of hypoglycemia in patients not treated with insulin.

The same conditional mechanism applies to the hemodynamic and renal effects in these patients. Notably, SGLT2 blockade reduces intraglomerular pressure and systolic blood pressure only when these values are elevated [25]. When blood pressure or filtration is already in a normal or low range, the modulated tubuloglomerular feedback loop is already “switched off”, and, therefore, no additional lowering occurs.

This self-limiting physiology explains why SGLT2i do not cause hypotension, hypoglycemia, or renal dysfunction in stable patients. When perfusion, glucose, and kidney function are within normal limits, these drugs become physiologically inert; when those parameters are excessive, these drugs can restore balance. For this reason, SGLT2i represent the safest and most adaptive component of GDMT, protective in stress and neutral in stability [26, 27].

How Can These Principles Be Applied in Daily Clinical Practice? SGLT2i can generally be prescribed to all patients with HF and an eGFR above 20 mL/min/1.73 m^2^. In the absence of active urinary infection, these agents are almost universally well-tolerated and should be included within standard GDMT.

## 6. Conclusion

Most deviations during HF GDMT reflect physiological adaptation rather than harm. A low heart rate without syncope is safe; a creatinine rise of ≤50% is acceptable if the eGFR remains ≥25–30 mL/min/1.73 m^2^; true hyperkalemia is rare with preserved filtration. SGLT2is act only when glucose levels, pressure, or filtration are elevated, remaining inert under normal conditions. These laboratory and hemodynamic changes should not be viewed as transient abnormalities to be reversed, but as stable physiological adaptations that reflect effective neurohormonal blockade, reducing renal workload, sympathetic tone, and limiting adverse remodeling, thereby improving long-term prognosis [1, 2]. Therefore, understanding these mechanisms replaces fear with physiology, thereby allowing the full and confident use of this life-saving therapy.

## Abbreviations

GDMT, Guideline-Directed Medical Therapy; ACEi, Angiotensin-Converting Enzyme Inhibitors; ARB, Angiotensin Receptor Blockers; ARNI, Angiotensin Receptor–Neprilysin Inhibitor; MRA, Mineralocorticoid Receptor Antagonist; SGLT2i, Sodium–Glucose Cotransporter 2 Inhibitors; eGFR, Estimated Glomerular Filtration Rate; NSAIDs, Nonsteroidal Anti-Inflammatory Drugs; CKD, Chronic Kidney Disease; RAAS, Renin–Angiotensin–Aldosterone System.
